# The Value of CBCT-based Tumor Density and Volume Variations in Prediction of Early Response to Chemoradiation Therapy in Advanced NSCLC

**DOI:** 10.1038/s41598-017-14548-w

**Published:** 2017-11-07

**Authors:** Qiang Wen, Jian Zhu, Xue Meng, Changsheng Ma, Tong Bai, Xindong Sun, Jinming Yu

**Affiliations:** 1 Department of Radiation Oncology, Shandong Cancer Hospital Affiliated to Shandong University, Shandong University, Jinan, 250117 China; 2 Department of Radiation Physics, Shandong Cancer Hospital Affiliated to Shandong University, Shandong University, Jinan, 250117 China; 3 Shandong Academy of Medical Sciences, Jinan, 250001 China

## Abstract

The correlations between early responses and the variations in physical density and primary tumor volume (TV) according to cone-beam computed tomography (CBCT) during chemoradiotherapy for non-small cell lung cancer (NSCLC) patients were investigated. 54 patients with inoperable and locally advanced NSCLC were included in this study. The CT numbers (CTN) and TV were measured on each of the seven observation points. The changes in the mean CTN values and the variation ratios of TV during the treatment course were analysed and correlated with the clinical outcomes, as evaluated by the RECIST criteria. For patients who responded to treatment, the CTN and TV change ratio decreased by 28.44 ± 13.12 HU and 32.01% (range, 8.46–61.67%); these values were significantly higher than those in the non-responding patients, with 19.63 ± 8.67 HU and 23.20% (range, −15.57–38%) (p = 0.016, p = 0.048), respectively. The area under curve for the combination of CTN and TV was larger than either alone (AUC = 0.751, p = 0.002). The differences between response and non-response were most significant between Fraction 10 and Fraction 15 for CTN changes and between Fraction 5 and Fraction 10 for the TV regression ratio. The changes in CTN and TV obtained from CBCT images have the potential capability to predict an early response of NSCLC.

## Introduction

Non-small cell lung cancer (NSCLC) is the main subtype of lung cancer and accounts for 80% of all lung cancers. Approximately 80% of all NSCLC cases were diagnosed at an advanced stage due to non-specific recognized symptom at an early stage^1^. Chemoradiation therapy (CRT) could provide a survival benefit for inoperable or locally advanced patients, with a median overall survival ranging from 25–35 months^2,3^.

Currently, the Response Evaluation Criteria in Solid Tumors (RECIST) has been widely applied in the clinical setting and has been introduced in evaluation tumor response to anti-cancer therapy^4–6^, although some limitations are being recognized. First, changes in the longest axis of targeted lesion is the main evaluation criterion in RECIST, which ignores changes in other forms to a certain extent, such as the short axis and tumor volume^7,8^. These morphologic changes based on uni-dimensional measurements neglect the molecular changes and cannot effectively reflect biologic alternations in tumors, which may lead to inaccurate predictions of treatment response. Second, the RECIST criteria are generally performed two or three months after treatment, which might delay the detection of disease progression and recurrence.

With advances in computed tomography (CT) technology, quantitative image analysis could provide CT density (HU, Hounsfield Unit) measurements within a region of interest (ROI). Regarding the tumor density decrease, Yang *et al*. and Mayer *et al*. proposed a reliable quantitative method to assess tumor response and predict clinical outcomes^9,10^. Previous studies have correlated the variation in the CT number (CTN) with radiation-induced pulmonary toxicity^11^ and breast cancer metastasis to axillary lymph nodes^12^.

Imaging-based three-dimensional measurement of tumor size change might more accurately and directly assess the response during the course of treatment. There is evidence that a change in tumor volume could be detected as early as 3 weeks after gefitinib administration in lung cancer, but the World Health Organization (WHO) criteria and RECIST cannot identify responses in that timeframe^13^. Hou *et al*. demonstrated that the early reduction of gross tumor volume had a statistically significant difference (p = 0.045) between treatment outcomes in head and neck cancer patients^14^. Therefore, changes in tumor volume have the potential to be an early surrogate marker of tumor regression or progression.

Rescanning and replanning via periodic CT scanning are not ideal due to the time required and additional irradiation^15^. As part of image-guided radiation therapy (IGRT), kilovoltage cone-beam computed tomography (KV-CBCT) could provide the capability to monitor the tumor density changes and volume regression during the course of chemoradiotherapy. Therefore, this study proposed to investigate the correlations between chemoradiation treatment response and variations in primary CTN and TV by KV-CBCT in NSCLC patients and to determine the appropriate time points for prediction.

## Materials and Methods

### Patient eligibility

From February 2014 to April 2015, 61 patients with locally advanced stage III NSCLC diagnosed by cytology or histology were included in this study. The inclusion criteria were as follows: (1) no previous anti-cancer treatment; (2) standard evaluation before treatment included CT imaging of chest and abdomen and brain magnetic resonance (MR); (3) good Eastern Cooperative Oncology Group performance status (ECOG PS) of 0 to 2 and good lung function (DLCO ≥ 50% and FEV_1_ ≥ 50%); and (4) a visible tumor on CT. Stage was defined according to the American Joint Committee on Cancer (AJCC, 6^th^ edition) staging system. Three patients were excluded from our study due to small tumor size (<1 cm in diameter), and four patients were excluded with a PS score of 3. In total, 54 patients who received concurrent CRT and serial KV-CBCTs as part of conventional fractionation radiotherapy were included in our study.

The study was approved by the Research Ethics Committee of Shandong Cancer Hospital, China. All protocols and methods were in accordance with the guidelines and regulations. Informed consent was provided by all participants.

### Chemoradiation therapy and follow-up

The chemotherapy regimens included etoposide 50 mg/m^2^ on D1-5, D29-33 and 50 mg/m^2^ on days 1, 8, 29, 36 for cisplatin. Paclitaxel plus carboplatin was administered as paclitaxel 40 mg/m^2^ weekly and AUC 2 for carboplatin. Pemetrexed with cisplatin was given as 500 mg/m^2^ on day 1 every 21 days for 3 cycles and 75 mg/m^2^ on day 1 for cisplatin. Chemotherapy doses were modified on account of toxicity levels and blood counts. Additional 4-dimensional CT (4D CT) scans were utilized for respiratory motion and tumor delineation. Contouring gross tumor volume (GTV) was performed on each of the 10 respiratory phases of the 4D CT data set, and the GTVs were combined to obtain the internal target volume (ITV). Radiotherapy plans were generated on the Varian treatment planning system (Varian Medical System, Inc., Palo Alto, California, USA). The radiation dose was 60 Gy over 6 to 7 weeks, as a 2 Gy/fraction to the primary tumor, with 5 fractions per week.

Follow-up data were obtained from Shandong Cancer Hospital and Institute medical records. For clinical examinations, chest CT imaging was performed at the first month after treatment, with follow-up imaging every two or three months in the first year, then every six months in the second and following years. Brain magnetic resonance (MR) and bone scintigraphy were not compulsory and were only administered if clinically indicated. Clinical response was evaluated for all patients based on the RECIST criteria systems, and the therapeutic response was evaluated as complete response (CR), partial response (PR), stable disease (SD) or progression disease (PD).

### CBCT scan and image analysis

CBCT images of the chest area were acquired prior to radiation delivery as part of the standard radiotherapy course. We acquired CBCT images to assess the changes in primary tumor density and volume during the course of therapy. KV-CBCT projection images were obtained with a voltage of 110 kV, tube current of 20 mA and a F0 filter. The detector size and source to detector distance were 25 cm × 25 cm and 150 cm, respectively. The 2.5-mm contiguous axial images were transferred to the Varian workstation and reconstructed into a 250 × 250 × 200 mm field of view. Scatter correction and ring artefact correction were used for each serial CBCT image. As suggested by Altorjai *et al*., a window/level setting of −600/1000 HU was used for lung lesion contouring^16^. Contours of the primary tumor were obtained from the KV-CBCT, which were automatically generated by 3D Slicer (a free open-source software platform for biomedical imaging research, www.slicer.org) and modified manually if necessary by two independent radiation oncologists (SXD, with 25 years of experience, and MX, with 10 years of experience). The CBCT serials with corresponding contours were analysed using the open-source Imaging Biomarker EXplorer (IBEX) software on the 1^st^, 5^th^, 10^th^, 15^th^, 20^th^, 25^th^ and 30^th^ fractions (F1, F5, F10, F15, F20, F25 and F30), which supported the CTN and TV variation value of the primary tumor as the average and standard deviation or median with range. To minimize the variations in outlines generated in this study, CTN numbers less than −200 HU or greater than 400 HU were excluded from analysis. In the same condition, delineation did not dramatically influence the analysis because only changes in density and volume were investigated.

In order to ensure CTN stability, the CBCT scan equipment was calibrated monthly by CIRS062m. There was no dramatic or detectable change in the CT number, which meant that no variation in KV-CBCT resulted in CTN changes in NSCLC patients.

### Statistics analysis

All statistical analyses were performed using the SPSS Ver. 19.0 software for windows (IBM SPSS Inc, Chicago, IL, USA). The correlation between the CTN and volume changes for the primary tumor, and the correlation between the CTN change and radiation dose were analysed using linear analysis. Categorized characteristics were compared between two groups using a Chi-squared test, and continuous variables were compared using a t-test. To combine the changes in CTN and TV, a logistic regression model was employed that allowed discriminating between response and non-response. A t-test was used to determine whether the response was significantly different from the non-response. Receiver operator characteristics (ROC) curves were created to identify the most powerful predictive period and establish a cut-off value for distinguishing response patients from non-response patients. All p-values were two-sided at a significance level of 95%, and p < 0.05 was considered statistically significant.

## Results

### Patient characteristics

The demographics and disease characteristics of all 54 patients are summarized in Table 1. The median follow-up period in this trial was 16.3 months (range, 5.0–30.1 months). The median age of all cases was 62 years (range, 32–78 years). Patients aged less than 65 years old accounted for 75.9%. Thirty-three patients with stage IIIA were included, as were 21 stage IIIB patients.

**Table 1:** Patient demographics and treatment characteristics.

Characteristics	N (%)
Age	
Median (range)	62 (32–78)
Gender	
Male	30 (55.6%)
Female	24 (44.4%)
Smoking	
Yes	37 (68.5%)
No	17 (31.5%)
Stage	
III A	30 (55.6%)
III B	24 (44.4%)
ECOG PS	
2	11 (20.4%)
0–1	43 (79.6%)
Histology	
Squamous	32 (59.3%)
Adenocarcinoma	22 (40.7%)
Chemotherapy regimens	
Etoposide + Cisplatin	23 (42.6%)
Paclitaxel + Carboplatin	21 (38.9%)
Pemetrexed + Cisplatin	10 (18.5%)

*Abbreviations*: ECOG PS = Eastern Cooperative Oncology Group performance status.

### CTN and tumor volume variations

The mean CTN exacted from the first CBCT (before first time radiotherapy) was 63.21 ± 28.93 HU, and the mean CTN extracted from last CBCT (before last time radiotherapy) was 38.29 ± 19.91 HU. Among the 54 cases, 61.1% of patients decreased at least 20 HU, and 12 patients’ CTN reduction fluctuated by approximately 10 (10 ± 5) HU. The difference in CTN between the first and last fractions on KV-CBCT was statistically significant (p < 0.001). The corresponding primary tumor volume was reduced for most of the patients. The tumor size decreased from 84.94 cm^3^ (range, 19.96 cm^3^–218.37 cm^3^) to 60.26 cm^3^ (range, 14.25 cm^3^–147.29 cm^3^) from the start to the end of radiation. 26 patients displayed a regression of the tumor size of >30%. A significant change in tumor volume was detected between the 1^st^ and 30^th^ fractions among all patients (p < 0.001).

**Figure 1:**
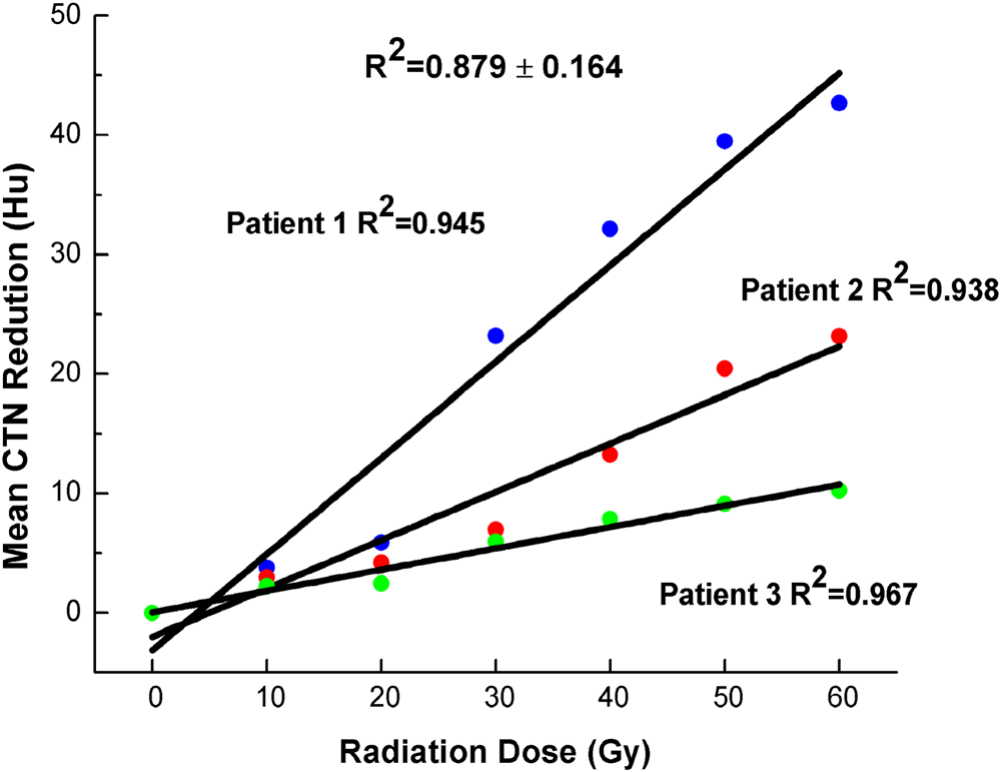
Details of correlation between mean CT number (CTN) changes and radiation dose delivery for three representative patients, randomly selected from 54 patients. The correlation data for all patients was R^2^ = 0.879 ± 0.164.

**Figure 2:**
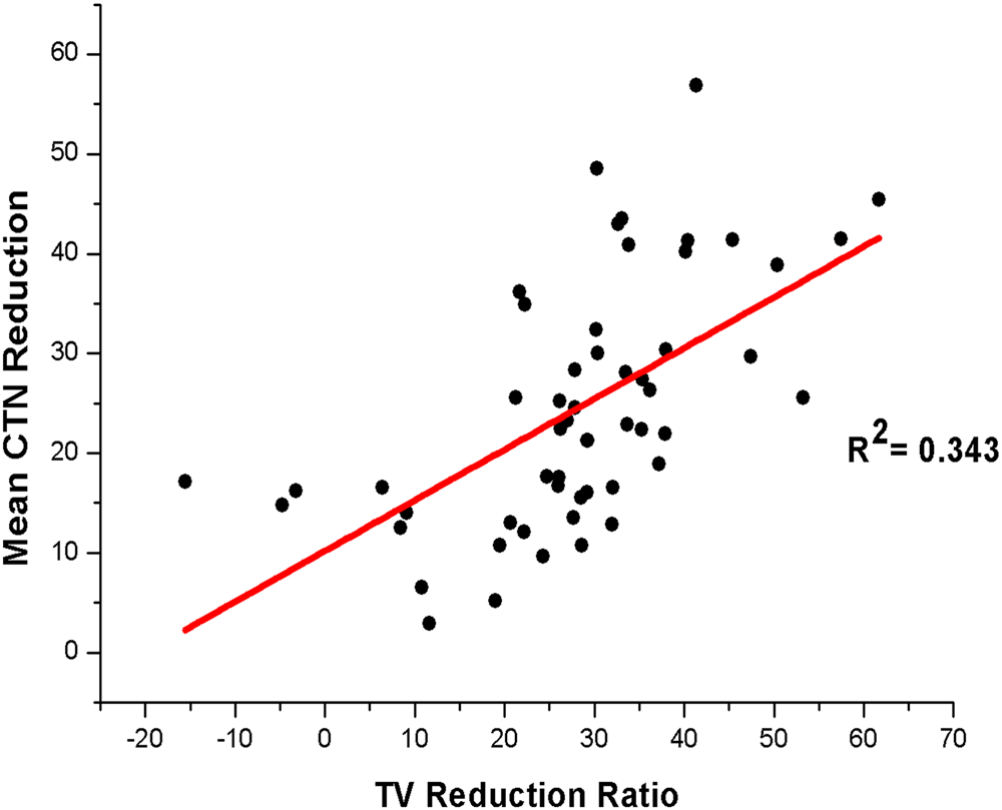
Correlation between mean CT number (CTN) changes and tumor volume (TV) reduction ratio for primary tumor among all patients. The correlation data for all patients was R^2^ = 0.343.

For all patients in the study, the mean CTN value reduction was significantly related to the radiation dose with R^2^ = 0.879 ± 0.164 and p = 0.002. The correlation data of three representative patients, randomly selected from the 54 patients, are presented in Fig. 1. The relationship between mean CTN value change and volume reduction ratio was R^2^ = 0.343 and p < 0.001 (Fig. 2).

### Prediction of CTN and tumor volume for response

Out of the 54 patients, 31 presented PR, 2 patients presented CR, 20 patients experienced SD and only one patient reported PD according to RECIST criteria.

The tumor clinical variables between the response group and non-response group are detailed in Table 2. In the univariate analysis of all factors, each characteristic was compared between the groups, and only CTN reduction and volume changes were associated with outcome. No other clinical variables had an effect on response. Although response rate tended to be lower in older patients (≥65 vs. <65 years, p = 0.055), this effect was still not significant. More than half of the patients (19/33) presented an objective response; their CTN value decreased above 25 HU. In the majority of the non-response patients (10/21), the CTN reduction fluctuated by approximately 10 (10 ± 5) HU. The mean densities of the primary tumor at baseline in the two groups were 67.62 ± 30.14 HU and 56.43 ± 25.52 HU, respectively. The CT density before radiotherapy was not significantly different between the groups (p = 0.149). However, after 60 Gy of conventional fractionation radiotherapy, the CTN variations were more significant in the response group (28.44 ± 13.12 HU vs. 19.63 ± 8.67 HU, p = 0.005).

**Table 2:** Univariate analysis of demographic and morphologic factors for NSCLC patients with response.

Characteristics	Response (33)	Non-response (21)	p-value
Gender			
Male	18	12	0.851
Female	15	9	
Age			
≥65	5	8	0.055
<65	28	13	
Smoking			
Yes	21	16	0.333
No	12	5	
Stage			
IIIA	19	11	0.708
IIIB	14	10	
ECOG PS			
2	6	5	0.617
0–1	27	16	
Histology			
Squamous	21	11	0.412
Adenocarcinoma	12	10	
Chemotherapy regimens			
Etoposide + Cisplain	16	7	0.491
Paclitaxel + Carboplatin	11	10	
Pemetrexed + Cisplatin	6	4	
CTN Baseline (HU)	67.62 ± 30.14	56.43 ± 25.52	0.149
CTN Change (HU)	28.44 ± 13.12	19.63 ± 8.67	0.005
Volume Baseline (cm^3^)	98.59 (19.96–218.37)	72.11 (32.01–182.39)	0.314
Volume Change ratio	32.01% (8.46–61.67%)	23.20% (−15.57–38.0%)	0.026

*Abbreviations*: ECOG PS = Eastern Cooperative Oncology Group performance status; CTN = computed tomography number.

The tumor volumes of the two groups at baseline did not differ before radiation therapy with p = 0.314. Nevertheless, an obvious heterogeneity of the change in tumor volume was observed in the radiotherapy procedure. The tumor volume in patients who had a response diminished by 32.01% (range, 8.46–61.67%), and patients who were categorized as non-response had tumor volumes that dropped by 23.20% (range, −15.57–38.00%) (p = 0.026). The patients whose primary tumor volume reduced by >50% were all from the response group, and the patients with regression ratio below 20% all belonged to the non-response group. In the multivariate analysis, the difference between the groups still showed statistical significance in the changes in CTN (p = 0.016) and TV (p = 0.048).

**Figure 3:**
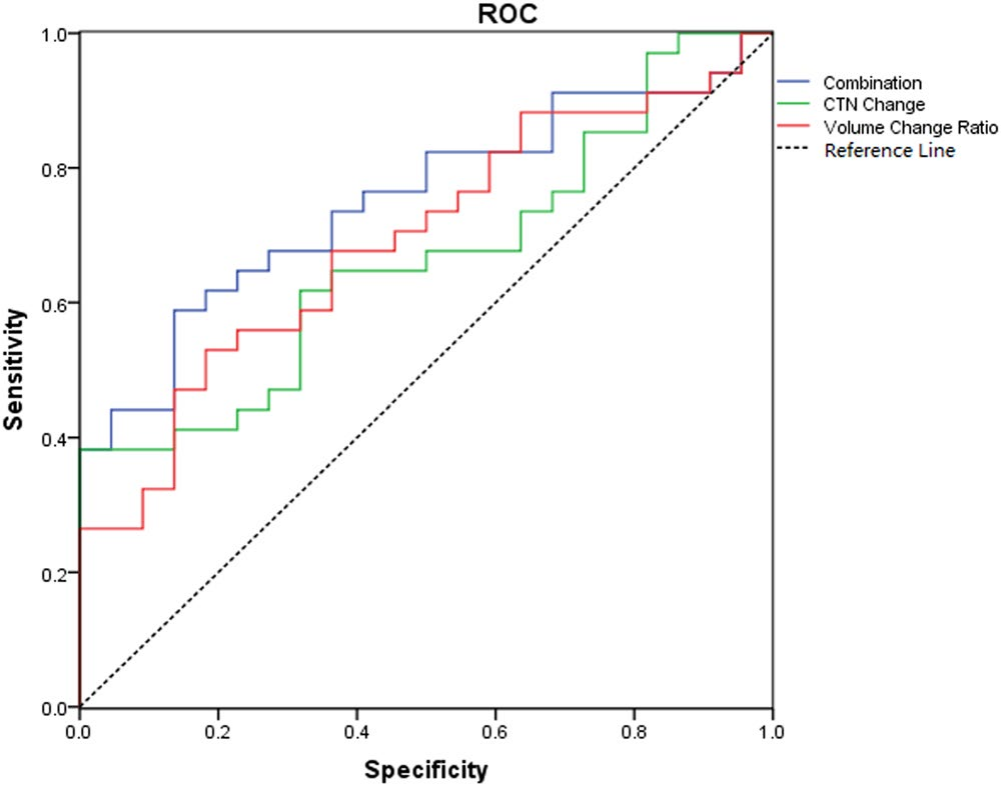
Receiver operating characteristic (ROC) curves for early response prediction. The area under the curve (AUC) for the models is shown in the figure.

This result illustrated that both CTN change and tumor volume regression could distinguish responding patients from non-responding patients, with p = 0.037 and p = 0.016, respectively (Fig. 3). Further, the logistic regression model and ROC analysis implied that the combination of change of CT density value and tumor volume had a higher AUC (AUC = 0.751) than CTN (AUC = 0.666) or tumor size (AUC = 0.693) alone for assessing treatment early response (p = 0.002). The sensitivity, specificity, positive predictive and negative predictive values were 58.8%, 86.4%, 87.16% and 57.17%, respectively.

### Factors effect on CTN and volume changes

Univariate analysis results demonstrated that both the CTN value and TV varied with histology. The change in the mean CTN in the squamous group (28.08 ± 11.28 HU) was much higher than that in the adenocarcinoma group (20.69 ± 12.40 HU) (p = 0.019). For the volume reduction ratio, we found that patients who were diagnosed as squamous (28.63%) had dramatically deceased volumes (p = 0.046), compared to the adenocarcinoma group (23.04%). However, we did not find any difference among the various chemotherapy regimen groups with the respect to CTN (p = 0.144) or volume (p = 0.213). In the multivariate analysis, the test showed that the histological type was an independent predictive factor for CTN reduction (OR = 1.554, 95% CI = 1.033–2.331, p = 0.035) but not for volume regression (OR = 1.320, 95% CI = 0.904–1.917, p = 0.141).

### Early response

**Figure 4:**
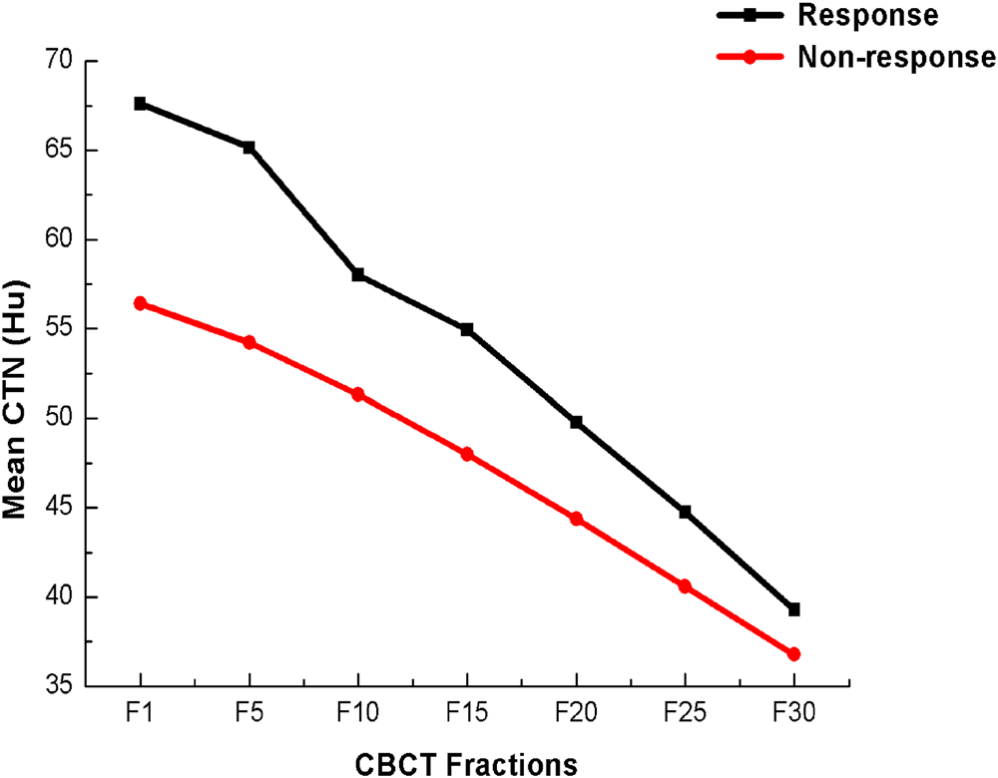
Tumor CT number (CTN) changes over radiotherapy (RT) course. The mean CTN changes in response group and non-response group were 67.62 ± 30.14 Hu and 56.43 ± 25.52 Hu.

CTN reduced gradually over the course of treatment. The curve for primary tumors is shown in Fig. 4. Major changes occurred at the early stage of radiotherapy, and the tumor density decreased more significantly in the first half of radiotherapy. The predictive value of the relating various slopes to treatment response were calculated by period, which illustrated that the mean HU decrease per Gy (ΔHU/Gy) of period Fraction 1- Fraction 10 (p = 0.094), Fraction 5- Fraction 10 (p = 0.001), Fraction 5- Fraction 15 (p = 0.004) and Fraction 10- Fraction 15 (p < 0.001) showed statistically significant associations with an early response. In the ROC analyses, the predictive value of Fraction 10-Fraction 15 was much higher than the other values (AUC = 0.753, p = 0.002), with the best cut-off value at 0.53 (Table 3). The sensitivity and specificity values were 58.8% and 90.9%, and the positive and negative predictive values were 90.6% and 58.3%, respectively.

**Table 3:** Comparisons between periodic slopes for response prediction in NSCLC patients.

	CTN (ΔHU/Gy)			Volume (ΔTV ratio/Gy)		
	TTEST	AUC	p-value	TTEST	AUC	p-value
**F1- F5**	0.665	0.531	0.7	0.364	0.571	0.374
**F1-F10**	0.094	0.678	0.026	0.083	0.628	0.046
**F1-F15**	0.149	0.592	0.247	0.377	0.548	0.546
**F5-F10**	0.001	0.75	0.002	0.019	0.667	0.036
**F5-F15**	0.004	0.721	0.006	0.121	0.596	0.339
**F10-F15**	<0.001	0.753	0.002	0.218	0.580	0.314

*Abbreviations*: AUC = area under curve; CTN = computed tomography number; F = fraction.

**Figure 5:**
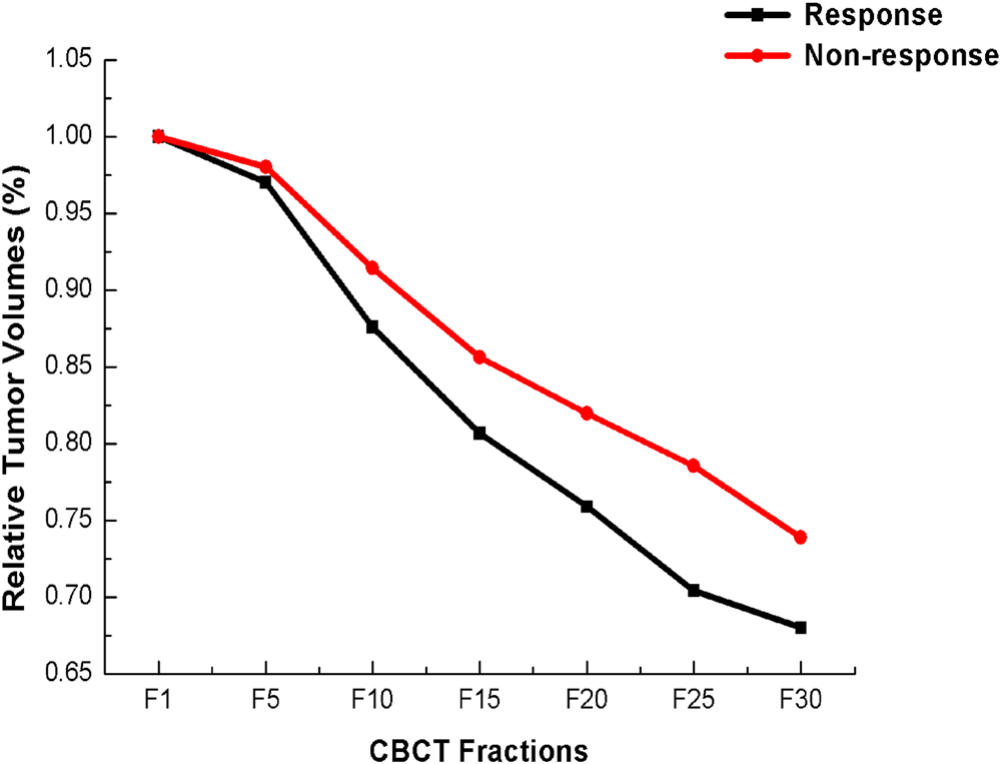
Tumor volume (TV) reduction ratios for patients with radiotherapy (RT). Tumor volume regressions in response group and non-response group were 31.76% ±14.78% and 26.12% ± 12.16%.

The volume change curve for primary tumors is displayed in Fig. 5, where the most of patients with a response presented a substantial regression in tumor volume between Fraction 1 and Fraction 15. As Table 3 indicates, the early regression of tumors was sufficient to predict a response in the period of Fraction 1- Fraction 10 (p = 0.083) and Fraction 5- Fraction 10 (p = 0.019). In the ROC analysis, Fraction 5- Fraction 10 (AUC = 0.667) was more powerful than Fraction 1- Fraction 10 (AUC = 0.628) (Table 3), with a cut-off value of 0.919. When we evaluated the change of TV for distinguishing the response from non-response, the overall sensitivity, specificity, positive and negative predictive values were 55.9%, 81.8%, 82.8% and 54.1%, respectively.

## Discussion

Currently, the predictors of NSCLC treatment response are varying and unstable, so a novel strategy is required to provide earlier and more accurate information about a patient’s prognosis. Most patients depend on a restaging chest and abdomen CT scan performed 4–8 weeks after the completion of therapy to determine the response results, which is a long waiting period, and the opportunity to adjust treatment plans may be missed. Through the identification of patients who will get benefit from treatment, patients may achieve equivalent results with lower RT doses and economic cost^17^. In this work, we investigated the possibility of using CTN and tumor volume regression ratio via CBCT images as predictors of treatment outcomes.

The CTN changes detected during the treatment process were specific to each patient; the apparent changes did not exist in all cases, which meant that the radiation dose was just one of the elements contributing to the CTN changes. In addition to histological types, there was a correlation between decreased CTN and radiosensitivity in patients with NSCLC^18^. Due to the presence of tumor cell heterogeneity, tumor cells might display different degrees of radiosensitivity when they have different pathological differentiation statuses. Radiation-resistant cells were more likely to survive radiotherapy, which contributed to the disparate degrees of CTN reduction. The mechanism for radiation-induced CT number changes was still indistinct and indefinite. The reasons for this are as follows. Cao *et al*. inferred that the radiation-induced tumor microenvironment improvement increased tumor blood volume (BV), which might lead to CT number decreases in GTV in this study^19^. It has been revealed that an increase in BV was detected in the primary tumor at an early course of radiotherapy and the patients with local control (5.1 mL/100 g) had statistically significantly higher BV than the patients with local failure (1.0 mL/100 g). According to that study, we concluded that the tumor volume began to shrink with the delivery of radiation doses, while the BV might increase. Therefore, tumor blood volume enhancement might result in the tumor density tending to be hypodense and might contribute to the CTN reduction. Another explanation might be that with an increasing radiation dose, the tumor began to shrink as a result of tumor cell apoptosis and led to CTN reduction. Third, it has been reported that CBCT CTN extracted during radiotherapy could predict radiation-induced lung injury (RILI). The potential RILI patients whose normal lung tissues densities surrounding lung cancer were higher than that in patients with non-RILI^20^. Consequently, RILI should be considered a factor that influences CTN reduction in further analyses. It is challenging to discriminate between recurrence and radiation-induced changes following inflammation by using CBCT. However, a study by Mattonen *et al*. demonstrated that quantitative alternations in HU and ground glass opacity (GGO) textural analysis could differentiate recurrence from RILI at 9 months post stereotactic radiotherapy (SBRT)^21^. Generally, more follow-up studies are necessary to verify this hypothesis and investigate the mechanism behind the complex phenomenon between the CTN changes and radiation doses.

Regarding tumor volume, the radiation modality and technique had an impact on size regression. Bosmans *et al*. suggested that there was no obvious shrinkage in the first 2 weeks of radiation therapy, with 1.8 Gy per fraction administered two times per day^22^. The reason for this finding might be that the treatment-induced inflammation counterbalanced lung cancer cell death. Hu *et al*. evaluated patients treated with SBRT, and their median TV regression reached only 15.91% on CBCTs^23^, which was much lower than those of patients with IMRT. A reasonable explanation for this was an insufficient treatment time for demonstrating a tumor volume decrease with SBRT.

Although both of CTN and TV received a similar radiation dose, the correlation between them was generally weak. Mahon *et al*. observed CTN reduction in 27 lung cancer patients treated with definitive CRT or radiotherapy alone and found that the rate of tumor volume was weakly correlated with HU change^24^. Furthermore, Feng *et al*. reported a weak correlation (r = 0.47) in neck and head cancer with radiation therapy^25^. As mentioned above, the radiation dose might be just one of the factors causing variations; more factors need to be discussed.

The correlation between a patient’s prognosis and variations in tumor density and volume has been widely investigated using CBCT and other imaging techniques. In previous studies, CTN was investigated in different tumor sites^24,26^. Compared to patients with CR, Vandecaveye *et al*. noticed that volume shrinkage of the first two weeks was much lower in patients with CRT resistance, with −2.8% ± 34.5 versus 31.3% ± 32.1 (p = 0.03)^27^. Bhide *et al*. suggested that the most significant regression of volume occurred in the second week of radiotherapy^28^. We herein indicate that serial CBCT images obtained during radiation-based therapy can predict the response.

Several previous studies also detected the heterogeneity of tumor volumes reduction for NSCLC by using various imaging modalities. Fox *et al*. assessed 22 patients with NSCLC using repetitive CT scanning and found a significant tumor shrinkage of 25% at 30 Gy and 44% at 50 Gy, which was larger than our median reduction of 28.28%^29^. The reason for this difference may be the intrinsic low soft-tissue contrast of the CBCT images, especially in differentiating the primary tumor from normal tissues. As Wang *et al*. suggested^30^, the low-contrast regions were inadequate to define the spatial transformation and volume reduction. Megavoltage computed tomography (MVCT) was also used to evaluate tumor reduction throughout radiotherapy in patients with lung cancer. Woodford *et al*. reported that daily MVCT was utilized to quantitatively assess response to helical tomotherapy at a total dose of 60–64 Gy in the conventional fraction and found GTV regression greater than 30% at any point before 40 Gy, which could dramatically improve the therapeutic ratio^31^. Our results, which were slightly different from those of Woodford *et al*., revealed that the volume change could not be detected as a gradual linear decrease. Furthermore, we noticed that some patients experienced an increasing tumor volume in the first week or the last period of treatment. The reasons for these might be due to high tumor proliferation and radiation-induced inflammation in normal lung tissues around the tumor. In contrast to our study, Brink *et al*. showed that significant TV shrinkage was related to poor overall survival and locoregional control using CBCT^32^, implying that a large regression during radiotherapy could be a surrogate biomarker of tumor aggressive. Considering the instability of the tumor volume, we not only estimated tumor regression but also monitored the change in CT density. Moreover, the seven sets of CBCT images used in our study could provide more data to specifically assess tumor changes in comparison to the work of Brink *et al*. More importantly, we determined appropriate time points for early response prediction by using week-to-week CBCT data.

Decreased tumor density and TV have been used to distinguish between responders and non-responders in gastrointestinal stromal tumor (GIST), as Choi *et al*. proposed^33,34^. They showed that a ≥15% change in tumor density and a ≥10% regression of tumor size were associated with better PFS than RECIST criteria. Compared to small cell lung cancer (SCLC), NSCLC is less sensitive to therapy. Anatomical changes might not manifest initially, even if the therapeutic paradigm is appropriate. Accordingly, TV assessment alone had a smaller AUC than the combination of biological and anatomical evaluation. Due to the multiplex factors effects mentioned above, especially the intrinsic limitation of CTN accuracy, CTN must be combined with TV change to predict early response.

Compared to RECIST, our criteria had two advantages for evaluating early treatment response. First, the CTN had the potential ability of assessing the internal structure changes of the tumor prior to morphological changes. Second, by combining the CTN and TV changes, the treatment response could be predicted with higher specificity and sensitivity than when using the long axis only. We noticed that the change in the long axis was much smaller than the volume change. In other words, the RECIST diameters may remain relatively stable while regression has occurred in other directions, as noted by McNitt-Gray^35^.

The present study had some limitations. First, a relatively small sample number was included. Future studies with larger numbers of patients are necessary to validate our results. However, our cases represent a study with regular regimens. Second, the accuracy of the values was influenced by the different slice thicknesses used in CT and CBCT reconstriction and by the potential limit of CBCT in soft-tissue contrast compared with CT, which could result in uncertainties^36^. A slower gantry speed or Monte Carlo-based scatter correction may improve the CBCT image quality^37,38^. As Mazzola *et al*. showed, another limitation was that the CBCT CT number may not represent the real HU due to various artefacts, including body scattering^36^. However, the utility of delta CT number could reflect the actual biological changes of tumors and minimize the interference from influencing factors, especially when we considered that the image acquisition conditions were consistent among serial CBCT. In terms of the CT number, Jin *et al*. suggested that the difference between CT and the first CBCT was not significant from −200 HU to 500 HU^39^. Similar findings were detected by Altorjai *et al*., who found that the grey value distribution histogram from CBCT was matched to the HU distraction histogram of CT in a fixed window with grey level of −200 and a width of 600^16^.

## Conclusions

The changes of CTN and TV obtained from CBCT images have the potential capability to predict the early response of NSCLC. Fraction 10 -Fraction 15 (CTN) and Fraction 5 - Fraction 10 (TV) were two appropriate time points for early response prediction in advanced NSCLC.
